# Genetic Variation in the Feeding Behavior of Isofemale Lines of *Nesidiocoris tenuis*

**DOI:** 10.3390/insects11080513

**Published:** 2020-08-07

**Authors:** Milena Chinchilla-Ramírez, Meritxell Pérez-Hedo, Bart A. Pannebakker, Alberto Urbaneja

**Affiliations:** 1 Unidad de Entomología UJI-IVIA, Centro de Protección Vegetal y Biotecnología, Instituto Valenciano de Investigaciones Agrarias (IVIA), CV-315, Km. 10,7, 46113 Moncada (Valencia), Spain; mperezh@ivia.es (M.P.-H.); aurbaneja@ivia.es (A.U.); 2 Wageningen University & Research, Laboratory of Genetics, Droevendaalsesteeg 1, 6708 PB Wageningen, The Netherlands; bart.pannebakker@wur.nl

**Keywords:** Miridae, zoophytophagous predator, phytophagy, zoophagy, biological control, tomato

## Abstract

**Simple Summary:**

Breeding of species for the benefit of humans is a well-known practice in plants and some vertebrates. However, its application to invertebrates, specifically biocontrol agents, is rather new and there is still a lack of information on breeding parameters of key traits. In this study, we address two important traits for biocontrol, i.e., the phytophagy and zoophagy of a population of the zoophytophagous predator *Nesidiocoris tenuis*. Here, we determine whether there is variation in these two traits and the proportion of that variation that has a genetic basis. Our results revealed the presence of genetic variation in both traits for this population. Genetic variation observed in zoophagy was larger than that found in phytophagy. These findings are relevant to the field of genetic improvement of biocontrol agents, since it reports quantitative values necessary for potential breeding programs. Moreover, our results suggest that if such variation is maintained in larger populations, it might be possible that selection for more zoophagous individuals could entail a reduction of the damages derived from its phytophagy. Furthermore, this study sheds light on genetic tools that could be applied to improve the biocontrol practice and, consequently, contribute to a more sustainable agriculture.

**Abstract:**

Zoophytophagous predators provide biocontrol services in various major crops of modern horticulture due to the combination of its predatory capacity and the induction of plant defenses derived from its phytophagy. However, under certain conditions of prey scarcity, these natural enemies can inflict plant damage. Exploitation of genetic variation and subsequent selective breeding on foraging traits is a potential alternative to overcome this inconvenience. In this study, we quantified the genetic variation of phytophagy and zoophagy of *Nesidiocoris* *tenuis* (Reuter) (Hemiptera: Miridae), a zoophytophagous predator widely used in tomato crops to suppress key pests. We compared nine isofemale lines on their capacity to produce necrotic rings and wilting on tomato plants as a proxy for phytophagy, as well as their efficacy to prey on *Ephestia kuehniella* Zeller (Lepidoptera: Pyralidae) eggs, as a proxy for zoophagy. Differences between isofemale lines in phytophagy and zoophagy indicated a genetic basis. Variation found in the zoophagy levels was larger than that in phytophagy levels. Our results showed that there is a genetic basis for the variation observed in the feeding behavior of isofemale lines of *N.* *tenuis*, highlighting the potential importance of selective breeding for such traits of biocontrol interest.

## 1. Introduction

The importance of zoophytophagous species to suppress pests in agroecosystems has increased over the last decades [1,2,3,4,5,6,7,8]. However, the assessment of genetic variation on traits of biocontrol interest in these species is rather recent [9,10]. Dumont et al. [9] tested the hypothesis that zoophytophagous populations consist of a mix of specialized genotypes (i.e., zoophagous, phytophagous, and generalists) instead of only one highly plastic genotype. In this study, Dumont et al. [9] demonstrated genetic differences in the feeding behavior of *Campylomma verbasci* (Meyer) (Hemiptera: Miridae), specifically in the zoophagy of the two different prey species this zoophytophagous feeds upon in apple orchards. These results shed light on the possibility to explore and exploit intraspecific genetic variation of interesting biocontrol traits in commercially available zoophytophagous species.

*Nesidiocoris tenuis* (Reuter) (Hemiptera: Miridae) is a cosmopolitan zoophytophagous predator that is extensively used in tomato crops in the Mediterranean basin to control different key pests [6]. Its major contribution in the biocontrol programs in tomato is attributed to its efficacy against the ubiquitous whitefly *Bemisia tabaci* (Gennadius) (Hemiptera: Aleyrodidae) and the invasive South American pinworm *Tuta absoluta* (Meyrick) (Lepidoptera: Gelichiidae) [3,4,5,6,11,12,13,14,15]. However, its status as a biocontrol agent is sometimes controversial because of the damage it can inflict in plant tissues when the prey levels decrease [6,16,17,18,19,20]. The exploitation of the genetic variation in the feeding behavior of *N. tenuis* was suggested as a means to improve its predatory efficiency, while mitigating the detrimental effects derived from its phytophagy [10].

Most natural enemies currently used in augmentative biological control programs are selected based on differences between species (interspecific variation) to effectively control pests. Nevertheless, there is a growing interest in the exploitation of genetic differences in the traits of interest within species (intraspecific variation) of natural enemies [21,22,23,24,25]. This approach was applied before in predatory insects, predatory mites, and parasitoids, with successful selection on traits such as pesticide resistance, increased fecundity, host preference, sex ratio, and improved climatic tolerance [26,27,28,29]. However, only few of these examples went beyond laboratory or pilot tests, and most of them were not continued after a few years, despite their positive outcomes. Financial, technical, and legal limitations were deemed as likely causes preventing the development of genetic improvement in biocontrol agents [25,28]. Therefore, the current efforts are focused on the generation of knowledge and optimization of procedures that was missing for arthropods, to promote artificial selection on biocontrol agents [23,24,25].

A first step for selection to be feasible is the presence of variability in the target trait, but more relevant is the existence of a genetic basis for at least part of the variation observed in that trait [28,30]. Once the genetic variation is explored and confirmed, multiple genetic tools could be further applied to identify the gene(s) and the factors involved in their expression on a certain population, and to choose the optimal approach for artificial selection [21]. For instance, the recent sequencing of the genome of *N. tenuis* [31] is one of those tools that enables the genetic exploration of this species for improvement of its biocontrol traits.

In this study, we aimed to investigate whether there is genetic variation in two important traits of the feeding behavior of a wild population of *N. tenuis*, i.e., phytophagy and zoophagy, by using an isofemale line approach. Necrotic rings and wilting inflicted in plant tissue, as well as consumption of *E. kuehniella* eggs, were used as a proxy for phytophagy and zoophagy, respectively. We expected to find variation among isofemale lines for phytophagy and zoophagy, indicative of genetic variation for these traits. Our study was the first to investigate the genetic basis of the phytophagy and zoophagy of *N. tenuis*, and the results shed light on the potential to use the genetic variation in these two traits to enhance the biocontrol services of this zoophytophagous predator.

## 2. Materials and Methods

### 2.1. Isofemale Lines and Plants

Wild adults and nymphs of *N. tenuis* were collected with an aspirator from two outdoor tomato farms located in Peñíscola, Castellón (Spain) (Field-1: 40°23′39.33″ N/0°24′24.79″ E; Field-2: 40°22′44.5″ N/0°23′49.71″ E) (ca. 2 km distance between fields) in September of 2016. The tomato plants in these farms were grown organic and no previous releases of *N. tenuis* were done before the time of collecting, but wild *N. tenuis* was reported to appear in these crops every season. The province of Castellón is considered a transition zone between the two regions where mirid predators are used in tomato farms—the warmer Southern Spanish territories, where mostly *N. tenuis* is released in tomato crops for pest control, and the temperate Eastern Spanish territories, where *Macrolophus pygmaeus* (Rambur) (Hemiptera: Miridae) is the predominant species used in tomato crops for pest suppression [32]. Wild populations of *N. tenuis* were scarce during the season of 2016 and only a few insects (adults and nymphs) were captured (Field 1: *n* = 9, Field 2: *n* = 16), hence, it was necessary to mix them to secure the establishment of the laboratory colony with the maximum diversity available at the moment of collection. Individuals collected in both farms were brought to the laboratory, mixed, and placed in a plastic cage (30 × 30 × 30 cm) with green bean pods (*Phaseolus vulgaris* L.) and eggs of *Ephestia kuehniella* Zeller (Lepidoptera: Pyralidae) provided twice a week as oviposition substrate and food source, respectively. This colony was kept in the laboratory during five generations at 25 ± 2 °C, 50% ± 10% RH, and 14L:10D h photoperiod (1 generation~22 days at these experimental conditions). Individuals from the colony were allowed to randomly mate and increase the population numbers necessary to found the isofemale lines. After five generations in the colony, the isofemale lines were initiated as follows—one male and one virgin female, both less than three-days-old, were allowed to mate in a Petri dish with mesh on the lid for ventilation (90 mm on diameter), with an entire green bean pod (ca. 15 cm long) as oviposition substrate and *E. kuehniella* eggs *ad libitum* supplied twice a week until the female died. Fifteen isofemale lines were founded but only nine of them survived (extinct isofemale lines did not survive beyond generation three). Each bean pod bearing eggs laid by the founder females was then placed in an individual muslin cage (25 × 25 × 25 cm), with new bean pods and *E. kuehniella* eggs. Insects of the same isofemale line were allowed to mate randomly but the parents and progeny were kept separated at all times by removing the bean pods bearing the laid eggs twice a week and placing them in a new cage. *E. kuehniella* eggs were supplied twice a week as food source. The isofemale lines were kept at 25 ± 2 °C, 50% ± 10% RH, and 14L:10D h photoperiod. Frozen *E. kuehniella* eggs for all rearings and experiments were provided by Koppert Biological Systems (Almería, Spain).

Tomato plants cv Raf Supermarmande (Mascarell Seeds, Spain) used in this experiment were in vegetative stages V5 to V7 (ca. 30–40 cm height). Plants were grown in pots (8 × 8 × 8 cm) filled with soil and vermiculite (3:1) and watered every two days. Plants were kept in pest-free climatic chambers until the start of the experiment, at 25 ± 2 °C, 50% ± 10% RH, and 14L:10D h photoperiod. This cultivar was known to be susceptible to the feeding damage of *N. tenuis*, hence, it was selected for this study to allow for the visualization and measurement of the phytophagy parameters explained below.

### 2.2. Phytophagy Experiment

The isofemale line approach is convenient to study the genetic variation in traits of interest. It consists of the establishment of several strains or lines, each from a single mated female, and the quantification of the trait in several offspring produced by each female [33,34]. Considering that rearing and experimental conditions for all isofemale lines are identical, the variation observed between them for the mean values of the trait could be considered of genetic origin [30,35]. This experiment was carried out in generation 11, due to the limited availability of individuals in the earlier generations that prevented the attainment of the minimum of replicates necessary, and to secure homozygosity in the isofemale lines, which increases with the generations in the isofemale lines [36].

As a proxy for phytophagy, we quantified the number of necrotic rings and wilting percentage produced by ten adults of each isofemale line per replicate, on two types of leaves of a tomato plant. Adult insects used in this experiment were three-five days-old. The age of the experimental individuals was chosen to allow sufficient time for the females to mature, mate, and oviposit, in order to secure enough progeny and survival of the isofemale lines for the following zoophagy experiment (see below). The phytophagy parameters were evaluated in leaves attached to entire plants. During the experiment, the plants were placed in trays inside a pest-free climatic chamber and watered every two days. The space between plants in the trays was enough to avoid differences in development or leaf quality due to competition for light, as well as to reduce the possibility of plant injuries during manipulation of the plants and trays. Provided that the apical part of the tomato plant is preferred by *N. tenuis* [17,37], the two youngest fully developed leaves in the upper section of the plants were selected for this experiment, and classified according to its position from the apical bud—the *young* leaf was the closest to the apical bud and the *old* leaf was the second-to-last from the apical bud. The apical bud was not used to test for phytophagy because of (1) the heterogeneity in their size and structure and (2) limitations on the maximum number of individuals available in each isofemale line, at a given generation. Each leaf was enclosed in a muslin bag (15 × 21 cm) and ten adults of the corresponding isofemale line were placed inside the muslin bag without a supplementary food source or prey. The insects were allowed to feed for five days. The dead individuals were removed daily from the muslin bags to prevent necrophagy during this experiment, and mortality per isofemale line was recorded. Dead individuals were not replaced with new individuals. After the feeding period, the remaining insects were removed, and the level of phytophagy was evaluated. Necrotic rings visible in the petiole and rachis were recorded. For wilting evaluation, each leaflet (five leaflets per leaf, all of similar size) was assigned the percentage of wilting in a 20% of the total leaf surface.

This experiment was carried out in ten consecutive blocks (all with individuals from generation 11, after the start of the isofemale lines). In each block, all isofemale lines were included with a minimum of one replicate per isofemale line (except isofemale lines 5 and 10 in blocks 6 and 7, respectively) (Table S1). A replicate consisted of one leaf enclosed in a muslin bag with ten adults (five males and five females). A total of 240 replicates were used in this experiment, with the number of replicates tested per isofemale line ranging between 21–33. One tomato plant always contained two replicates, one on the *young* leaf and one on the *old* leaf, and replicates on the same plant never belonged to the same isofemale line to exclude any possible plant effect. Each block consisted of similar-aged adults (three-five days old) and plants of similar developmental stage, as described above. Similar-aged adults were obtained from the bean pods, which were replaced in the cages twice a week, as previously described. This experiment was carried out in a pest-free climatic chamber at 25 ± 2 °C, 50% ± 10% RH, and 14L:10D h photoperiod.

### 2.3. Zoophagy Experiment

In a separate experiment, we quantified the number of *E. kuehniella* eggs preyed upon by the adults of each isofemale line for 24 h, as a proxy for zoophagy. This experiment was carried out in generation 15 because of the decline in the number of individuals in all isofemale lines after the phytophagy experiment, in which most adults of each isofemale line were used to achieve enough replicates. Isofemale line 5 was excluded from the zoophagy experiment because the number of individuals were not enough for the minimum of replicates needed for this experiment. A total of 193 individuals were used in this experiment, with 22–26 individuals tested per isofemale line. For this, females and males of *N. tenuis* less than five-days-old were starved for 24 h, with a moist cotton plug for water supply. After the starvation period, the insects were placed individually in a Petri dish (55 mm on diameter), and *E. kuehniella* eggs (*n* = 120) were offered to each individual in a piece of sticky cardboard. Insects were allowed to feed for 24 h. After the feeding period, the *N. tenuis* adults were removed and the number of preyed eggs was counted with the help of a dissecting microscope. Experimental conditions were 25 ± 2 °C, 50% ± 10% RH, and 14L:10D h photoperiod.

### 2.4. Statistical Analysis

The variation for necrotic rings, wilting, and zoophagy was analyzed in two steps. For necrotic rings, we first fitted a linear mixed-effect model (LMM) on the square-root-transformed data to estimate the genetic variation between isofemale lines. In this model, ‘leaf type’ (young or old) and ‘mortality’ were entered as fixed effects, and ‘block’ and ‘line’ (9 isofemale lines) were entered as random effects. This model estimated the variance components by REML, which allowed for the estimation of the broad-sense heritability (*H*^2^) in isofemale lines [34,38]. To analyze possible differences in the number of necrotic rings between the isofemale lines, a second LMM was fitted with ‘block’ in the random structure and ‘leaf’, ‘mortality’, and ‘line’ in the fixed structure. ‘Line’ fitted as a fixed effect allowed for the comparison of the number or necrotic rings between the isofemale lines.

Similarly, wilting percentage was also analyzed following these two steps. First, genetic variation was estimated with an LMM fitted on arcsine-transformed data, with ‘leaf’ and ‘mortality’ as fixed effects, and ‘block’ and ‘line’ as random effects. The comparison of wilting percentages between isofemale lines was done by fitting a second LMM with ‘block’ as random effect and ‘leaf’, ‘mortality’, and ‘line’ as fixed effects. For zoophagy, genetic variation was estimated by fitting an LMM on square-root-transformed data, with ‘sex’ as fixed effect and ‘line’ as random effect. Comparison between isofemale lines was done after fitting a linear model on the square-root-transformed-data with ‘sex’ and ‘line’ as fixed effects. Model selection was done on the basis of the Akaike Information Criterion (AIC); models with the lowest AIC were selected as the best-fitted models [39] (Table 1). Significant fixed effects were followed by multiple comparison between the isofemale lines with Bonferroni correction (α = 0.05). Finally, Spearman Rank Correlation Analysis was used to estimate the correlations between zoophagy, necrotic rings, and wilting percentage within the isofemale lines. All statistical analyses were performed in R (version 3.6.1) [40].

### 2.5. Heritability

As an estimate of the genetic variance, the broad-sense heritability (*H*^2^) was calculated, as the ratio of the total genetic variance (V_G_) (i.e., additive, dominance, and epistatic) to the total phenotypic variance (V_P_) [38] (Table 2). The ratio V_G_/V_P_ expresses the extent to which the phenotype of an individual is determined by its genotype, hence, it is also known as the degree of genetic determination [38]. For necrotic rings and wilting, V_G_ was represented by between-line variance and V_P_ was represented by the sum of between-line variance and environmental variance (i.e., block variance and residual variance). For zoophagy, V_G_ was represented by between-line variance and V_P_ was represented by the sum of between-line variance and within-line variance. Additionally, we calculated the coefficient of genetic variation (V_G_) for the phytophagy and zoophagy traits from the estimated genetic components, with the formula:

(1) $${\mathrm{CV}}_{G}\;=100*\frac{\sqrt{V_{G}}}{\;\bar{Χ}}$$

where V_G_ is the genetic variance and $\bar{Χ}$ is the trait mean. The coefficient of genetic variation (V_G_) is another parameter used as an indication of the ability of a population to respond to natural or artificial selection, i.e., the evolvability of a trait [41].

## 3. Results

### 3.1. Phytophagy

**Figure 1:**
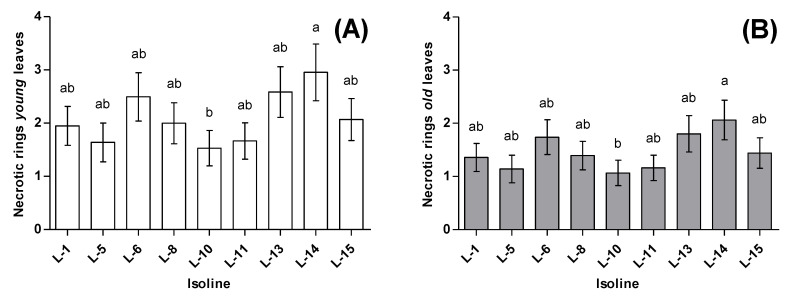
Average necrotic rings inflicted by *N. tenuis* on tomato plants. (**A**) Average necrotic rings in ‘young’ leaves, (**B**) Average necrotic rings in ‘old’ leaves. Bars sharing letters are not significantly different; error bars represent SE.

The number of necrotic rings was significantly different between the ‘old’ leaves and the ‘young’ leaves (F_1221_ = 17.86, *p* < 0.0001), with ‘young’ leaves showing more necrotic rings (2.1 ± 0.2) than ‘old’ leaves (1.4 ± 0.2). The number of necrotic rings inflicted on the tomato plants also differed across the isofemale lines (F_8221_ = 3.82, *p* = 0.0003). Isofemale line 14 inflicted the highest number of necrotic rings for both types of leaves, with an average of 2.9 ± 0.5 for ‘young’ leaves and 2.1 ± 0.4 for ‘old’ leaves. The isofemale line producing the least necrotic rings on both types of leaves was isofemale line 10, with an average of 1.5 ± 0.3 for ‘young’ leaves and 1.1 ± 0.2 for ‘old’ leaves (Figure 1). The broad-sense heritability for necrotic rings infliction was *H*^2^ = 0.16 (likelihood-ratio test: $\chi_{1}^{2}$ = 7.74, *p* = 0.005) (Table 2).

**Figure 2:**
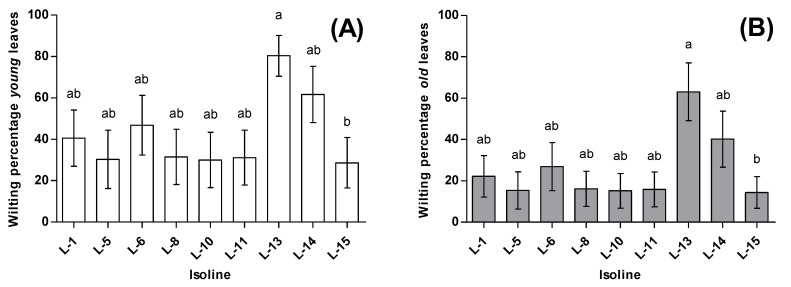
Average wilting percentages inflicted by *N. tenuis* on tomato plants. (**A**) Average wilting percentage in ‘young’ leaves, (**B**) Average wilting percentage in ‘old’ leaves. Bars sharing letters are not significantly different; error bars represent SE.

For wilting percentage, significant differences were observed between leaf types (F_1220_ = 27.78, *p* < 0.0001). In the ‘young’ leaves, the percentage of wilting reached an average of 42.3 ± 5.9%, whereas in the ‘old’ leaves, the wilting average was 25.5 ± 5.5%. Differences were also observed between the isofemale lines (F_8220_ = 518, *p* < 0.0001). Isofemale line 13 produced the highest wilting proportion on both ‘young’ and ‘old’ leaves (80.3 ± 9.8 and 63.1 ± 13.9, respectively), whereas the lowest proportion of wilted leaves was produced by isofemale line 15, with 28.6 ± 12.2 for ‘young’ leaves and 14.4 ± 7.7 for ‘old’ leaves (Figure 2). Mortality showed a significant effect on the wilting percentage (F_1220_ = 15.96, *p* = 0.0001), with the highest observed mortality in isofemale line 8 (5.79 ± 0.35) and the lowest in isofemale line 15 (2.85 ± 0.36). The estimated broad-sense heritability for wilting was *H*^2^ = 0.18 (likelihood-ratio test: $\chi_{1}^{2}$ = 7.45, *p* = 0.006) (Table 2).

### 3.2. Zoophagy

**Figure 3:**
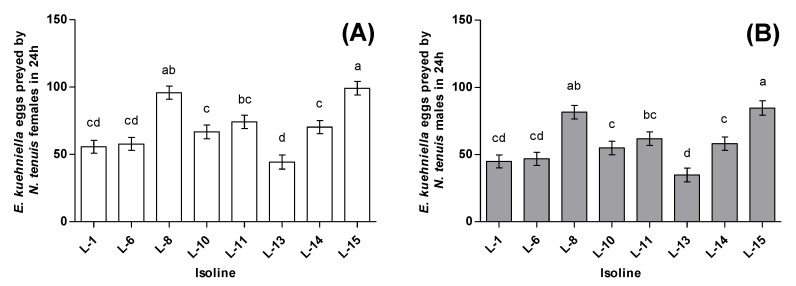
Average *E. kuehniella* eggs preyed by *N. tenuis* in 24 h. (**A**) Average *E. kuehniella* eggs preyed by *N. tenuis* females (**B**) Average *E. kuehniella* eggs preyed by *N. tenuis* males. Bars sharing letters are not significantly different; error bars represent SE.

The consumption of *E. kuehniella* eggs in 24 h significantly differed between sexes (F_1184_ = 24.18; *p* < 0.0001). For females, isofemale line 15 showed the overall highest predation rate with 99.2 ± 5.0 eggs preyed in 24 h, whereas isofemale line 13 preyed the lowest rate with 44.3 ± 5.2 eggs (Figure 3A). In the case of males, the highest predation was also observed in isofemale line 15 (84.7 ± 5.4), whereas isofemale line 13 showed the lowest predation rate with 34.8 ± 5.1 eggs in 24 h (Figure 3B). The amount of *E. kuehniella* eggs preyed upon also differed across isofemale lines (F_7184_ = 14.45; *p* < 0.0001) (Figure 3A,B). Broad-sense heritability estimate for zoophagy was *H*^2^ = 0.37 (likelihood-ratio test: $\chi_{1}^{2}$ = 57.57, *p* < 0.0001) (Table 2).

**Figure 4:**
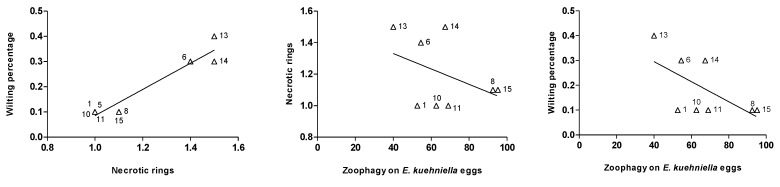
Correlation between wilting percentage and necrotic rings, and necrotic rings and zoophagy on *E. kuehniella* eggs, and wilting percentage and zoophagy on *E. kuehniella* eggs by *N. tenuis* on tomato leaves. Numbers represent the isofemale lines ID.

The number of necrotic rings was positively correlated with the wilting percentage (Spearman’s rho = 0.46, *p* < 0.0001) (Figure 4). Wilting percentage and zoophagy were negatively correlated (Spearman’s rho = −0.19, *p* = 0.0041) and there was no significant correlation between the necrotic rings and zoophagy (Spearman’s rho = −0.06, *p* = 0.3442).

## 4. Discussion

The presence of genetic variation in a trait is fundamental for selection processes to take place. In biocontrol agents, the existence of natural genetic variation in traits of interest, provides the opportunity to select for lower or higher values in those traits that could be useful in the biocontrol practice [21,24,25,28,29]. To the best of our knowledge, this is the first study to quantify the genetic variation in the feeding behavior of isofemale lines of *N. tenuis*. Our study adds to the growing body of literature reporting quantitative values of genetic variation, needed to enable successful genetic improvement of the biological control agents [29]. The phytophagy experiment showed that the ability of *N. tenuis* to produce necrotic rings and wilting on tomato plants differed across the isofemale lines and the leaf types. Similarly, differences were also observed for zoophagy across isofemale lines in their consumption of *E. kuehniella* eggs.

Necrotic rings in the stems and petioles of tomato plants are the most visible injuries produced by *N. tenuis* [6]. Our results showed that these injuries were more abundant in ‘young’ leaves than in ‘old’ ones. Similarly, wilting percentage on ‘young’ leaves was higher than that of ‘old’ leaves. This is consistent with previous studies that reported a higher number of necrotic rings in younger and softer plant tissues [37,42]. Thus, this suggest that the differences in the level of damage observed between leaf types are likely related to a gradient in tenderness or susceptibility of the leaf tissues. However, an experiment on the feeding behavior of the isofemale lines in different plant tissues is necessary to confirm this, since morphological differences (e.g., trichomes, exudates) could also influence the predator behavior, and thus the damage level. Interestingly, the phytophagous behavior of the isofemale lines was consistent across the two types of leaves for the two phytophagy parameters evaluated, i.e., isofemale lines inflicting more damage in ‘young’ leaves were also the isofemale lines inflicting more damage in ‘old’ leaves. Moreover, the positive correlation between necrotic rings and wilting also revealed consistency in the damage inflicted per isofemale line, with the isofemale lines inflicting more necrotic rings as the isofemale lines also cause more wilting, thus, reinforcing the presence of an isofemale line effect. Additionally, this positive correlation suggest that the occurrence of necrotic rings likely affects the transport of water and nutrients, thus, causing more wilting in tissues with higher number of necrotic rings.

Our results showed variability in the levels of necrotic rings and wilting percentage inflicted across the isofemale lines. However, the genetic component in both of these phytophagy parameters is rather limited, as suggested by the low *H*^2^ values. Besides the drawbacks commonly experienced by isofemale lines maintained for a number of generations in the laboratory, such as genetic drift, [36], other factors specific to zoophytophagous species can also explain low genetic variation in some of their traits. Castañé et al. [42] pointed at the complex interaction of physiological, behavioral, and morphological aspects of the insect, the host plant, and the environment that leads to the damage occurrence when zoophytophagous mirid predators are present.

For instance, as in many hemipterans, *N. tenuis* is a zoophytophagous insect that needs plant tissue for both feeding and oviposition [43]. Phytophagy is regarded as essential for extraoral digestion and survival of zoophytophagous species rather than facultative [42], and securing a favorable environment for their offspring is fundamental for several species [44], especially, when the eggs are inserted in the plant tissue, as is the case for *N. tenuis*. Thus, it is possible that the low genetic variation observed in the phytophagy-related traits is a trade-off resulting from selection acting on other related traits [45], such as reproduction. In addition, low heritability is consistent with strong selection pressure [46]. It is suggested that behavioral traits might be under the same type of selection as life history traits [47], whose heritability values are low often times. Thereby, the *H*^2^ values observed for phytophagy might be an indication of strong selection controlling this specific feeding strategy. Furthermore, genetic variation of species associated with cropping systems tends to be limited as a consequence of a homogeneous habitat (i.e., monocultures) that favors certain genotypes of the populations [48]. The individuals for our initial population were collected from tomato farms, where plant resources are homogeneous and readily available through the year. Hence, the individuals collected could be those genotypes already adapted to better exploit plant resources, thus, preventing the capture of a wider diversity for phytophagy traits. Nevertheless, the estimates of evolvability (CV_G_) for the phytophagy traits reveal that in the case of necrotic rings, the low *H*^2^ value could likely be the consequence of a larger environmental variance and not necessarily due to low genetic variance. Thus, some degree of response to selection might still be expected for the necrotic rings.

Zoophagy in *N. tenuis* is essential for its development [49,50]. In the present study, we found genetic variation for zoophagy among the *N. tenuis* isofemale lines. Larger differences between isofemale lines for this trait and the higher *H*^2^ values, suggest that a greater proportion of variation is due to variation in genotypes for zoophagy than in the phytophagy traits. This larger genetic variation is consistent with the conditions of constant fluctuations in prey densities and prey species faced by *N. tenuis* in cultivated systems. Other studies also argued the spatial and temporal changes in prey levels in the agricultural systems as an important cause for the genetic variation in other predatory invertebrates [9,51]. In addition, the larger variation and larger *H*^2^ values observed in this trait suggests that zoophagy is probably under less strong selection pressure [46], hence, allowing for faster responses to changing environments [51], such as those experienced in agroecosystems, in terms of prey. Interestingly, and opposite to the estimates for necrotic rings, the estimate of evolvability (CV_G_) for zoophagy is low, relative to a moderate value of *H*^2^. This is likely explained by a lower environmental variance relative to the genetic variance observed for this trait.

Previous studies with natural populations of *N. tenuis* showed that plant damage decreases when prey availability increases in tomato plants [52], and greenhouse experiments with commercial strains also showed a negative correlation between phytophagy and zoophagy [16,42]. In the present study, the correlation of phytophagous traits and zoophagy was negative, but it was significant only for the wilting percentage, not for the necrotic rings. However, there is a trend for the most zoophagous isofemale lines to be also the lines that inflict less damage. Further experiments with moving prey and other prey species in the plant would also be necessary to confirm whether this correlation is also maintained in more natural conditions. Interestingly, these negative correlations suggest that eventual selection for more zoophagous individuals in this population of *N. tenuis* could indeed entail a reduction in plant damage. However, in this study, *N. tenuis* did not have access to plant and prey simultaneously. A choice experiment would be necessary to confirm whether this increased zoophagy is indeed the result of diet specialization in certain isofemale lines (e.g., L-8 and L-15 zoophagy-specialized, and L-13 and L-14 phytophagy-specialized), or if it is due to individuals with increased voracity that can also perform well in absence/scarcity of prey. In the event that the increased zoophagy was due to diet specialization, breeding programs on these individuals could bring important benefits in terms of plant damage for the biocontrol practice. Nevertheless, such breeding attempts should also consider the potential drawback that zoophagy-specialized individuals might not survive periods of prey scarcity, which could mean an increase in the number of releases/introductions of this biocontrol agent in the crops.

The results of our study suggest an interesting potential of the genetic variation in the feeding behavior of *N. tenuis,* and of the isofemale lines approach, as a tool to be exploited in genetic improvement programs of biological control agents. Although the broad-sense heritability (*H*^2^) calculated on isofemale lines can sometimes lead to overestimations of the actual heritability in natural populations [36], the existence of a genetic component in feeding-related traits of a biocontrol agent is promising. The lower values of *H*^2^ and the smaller differences between isofemale lines observed in phytophagy, in addition to the diverse nature of the factors influencing its variation, suggest that this trait could be more challenging to target inbreeding programs. Although the evolvability (CV_G_) estimate observed for necrotic rings is still a good sign for potential selection against this trait and should be further investigated. Conversely, the larger variation observed in zoophagy represents a positive output for biocontrol improvement. The existence of isofemale lines showing higher predation rates could favor a decrease in the predator—prey ratio currently used in crops [10]. Hence, the use of fewer individuals of these “zoophagous” isofemale lines would allow a successful pest control, while reducing the exposure of crops to phytophagy and potential damage. It would be important to replicate these experiments with individuals collected in different environments and host plants, especially from non-agricultural settings, to increase the possibilities of capturing higher diversity of wild populations.

## 5. Conclusions

The presence of genetic variation in traits of biocontrol interest is fundamental for selection of improved biocontrol agents. Our study was the first to quantify the genetic variation of the feeding behavior of *N. tenuis*. Phytophagy-related traits might be more challenging to select against, due to the interaction of several factors, whereas the variation found for zoophagy-related traits is promising for the biocontrol practice. As such, our study adds to the growing body of literature reporting quantitative values of genetic variation, needed to enable the successful genetic improvement of biological control agents.

## Figures and Tables

**Table 1:** Akaike Information Criterion (AIC) for different linear mixed-effect models (LMM) for number of necrotic rings and wilting percentage inflicted on tomato plants (*n* = 240 replicates from 9 isofemale lines) and zoophagy (i.e., consumption of *E. kuehniella* eggs in 24 h) (*n* = 193 individuals from 8 isofemale lines) by *N. tenuis* adults. Values in bold indicate the model selected based on the lowest AIC.

Fixed Effects	Random Effects	Akaike Information Criterion (AIC)		
		Necrotic Rings	Wilting	Zoophagy
Leaf + Mortality	Block + Line	457.21	**304.56**	
Leaf + Mortality	Line	493.52	314.91	
Leaf + Mortality	Block	463.03	310.01	
Leaf	Block + Line	**451.19**	308.96	
Mortality	Block + Line	468.64	321.92	
Sex	Line			**714.31**
Sex				769.89

**Table 2:** Estimates of the means and standard errors (SE), genetic variation (V_G_), environmental variation (V_E_), broad-sense heritability (*H*^2^), and coefficient of genetic variation (CV_G_) for infliction of necrotic rings and wilting on tomato leaves, and zoophagy (i.e., consumption of *E. kuehniella* eggs in 24 h) by *N. tenuis*. Estimates are based on the squared-root-transformed data for necrotic rings and zoophagy, and arcsine-transformed data for wilting.

Parameter	*n*	Mean ± SE *	V_G_	V_E_	*H* ^2^	CV_G_ (%)
Necrotic rings	240	1.177 ± 0.044	0.070	0.444	0.16	22.54
Wilting	240	1.254 ± 0.032	0.039	0.217	0.18	15.77
Zoophagy	193	7.961 ± 0.131	1.190	3.231	0.37	13.70

* Units: necrotic rings (number on leaves), wilting (percentage of leaf surface), and zoophagy (eggs of *E. kuehniella* consumed).

## Supplementary Materials

The following are available online at https://www.mdpi.com/2075-4450/11/8/513/s1. Table S1: Number of replicates per line in each block and per leaf type tested in the phytophagy experiment.
