# Supplementary material for: Genetic Variation in the Feeding Behavior of Isofemale Lines of Nesidiocoris tenuis
Source: Insects. 2020 Aug 7;11(8):513. doi: 10.3390/insects11080513 (PMC7469200; doi:10.3390/insects11080513)
Supplement: Supplementary file 1 [file insects-11-00513-s001.pdf]

Table S1. Number of replicates per isofemale line in each block and per leaf type tested in the phytophagy experiment. One replicate consisted of one leaf enclosed in a muslin bag with ten *N. tenuis* adults (five males and five females).

**Isofemale line**	**Leaf type**	**Block**										**Total replicates per leaf**	**Total replicates per isofemale line**
		**1**	**2**	**3**	**4**	**5**	**6**	**7**	**8**	**9**	**10**		
1	*Young*	2	1	2	2	–	3	3	1	2	1	17	33
	Old	1	1	1	1	1	4	5	1	–	1	16	
5	*Young*	1	–	1	1	1	–	1	3	2	1	11	21
	Old	2	1	1	1	–	–	–	2	–	3	10	
6	*Young*	1	1	2	1	–	1	2	3	2	2	15	28
	Old	2	1	1	–	1	1	2	3	1	1	13	
8	*Young*	2	1	–	1	1	2	2	3	–	1	13	30
	Old	2	1	1	2	1	2	1	3	2	2	17	
10	*Young*	2	–	2	2	1	–	–	1	2	2	12	23
	Old	1	1	1	1	1	1	–	1	3	1	11	
11	*Young*	1	1	1	1	1	1	1	3	1	2	13	27
	Old	2	–	–	2	2	1	1	3	2	1	14	
13	*Young*	1	2	–	2	1	1	2	1	2	1	13	26
	Old	2	–	1	1	1	1	2	1	2	2	13	
14	*Young*	2	–	1	1	1	1	1	3	1	1	12	26
	Old	1	1	2	2	1	–	–	3	2	2	14	
15	*Young*	2	1	1	2	1	1	–	3	2	2	15	26
	Old	1	1	1	1	–	–	1	3	2	1	11	
								**Total replicates of the experiment**					**240**
